# Developing recommendations for digital collaboration meetings across healthcare levels and services for patients with chronic pain- a qualitative study

**DOI:** 10.1186/s12913-026-14345-0

**Published:** 2026-03-11

**Authors:** Torunn Hatlen Nøst, Astrid Woodhouse, Wenche Ann Similä, Karen Walseth Hara, Tormod Landmark, Ann-Elise Havnen Solvang, Mari Glette, Aslak Steinsbekk

**Affiliations:** 1 Department of Mental Health, Faculty of Medicine and Health Sciences, Norwegian University of Science and Technology, Trondheim, 7491 Norway; 2 Clinic of Anaesthesia and Intensive care, Clinical Research Facility, St. Olavs Hospital, Trondheim University Hospital, Trondheim, Norway; 3 Department of Circulation and Medical Imaging, Norwegian University of Science and Technology, Trondheim, Norway; 4 Department of Public Health and Nursing, Faculty of Medicine and Health Sciences, Norwegian University of Science and Technology, Trondheim, Norway; 5 The Norwegian Labour and Welfare Service of Trøndelag, Trondheim, Norway; 6 Digital health care unit, Norwegian Centre for E-Health Research, Tromsø, Norway

**Keywords:** Chronic pain, Interprofessional collaboration, Patient-centred care, Qualitative research

## Abstract

**Background:**

Collaboration between healthcare services is essential for the quality and continuity of care for persons with chronic pain. Collaboration meetings using digital solutions can be useful in overcoming collaboration challenges. However, there is a scarcity of studies on conducting digital collaboration meetings across services, particularly across healthcare levels for patients with chronic pain. Therefore, the aim of the study was to develop recommendations for how to plan and conduct digital collaboration meetings across care levels and services for patients with chronic pain.

**Methods:**

A qualitative study collecting data from a development process for digital collaboration meeting recommendations was conducted between October 2022 and June 2023. Data were collected at workshops, discussions in the clinic and research group meetings. The analysis focused on topics that generated the most discussions during the development process.

**Results:**

The discussions leading to the recommendations were categorised into three themes: ‘Patient-centeredness in the digital collaboration meeting’, ‘A common action plan’ and ‘Good meeting climate’. The main discussions included what it meant to place the patient at the centre of the activities, whether the patient should be present when professionals discussed the interprofessional assessments, whether clinical notes and suggestions for action should be distributed before the digital collaboration meeting, and how to ensure well-led meetings with clear agendas.

**Conclusions:**

A set of recommendations for how to plan and conduct digital collaboration meetings across care levels and services for patients with chronic pain was developed. Due to practical issues and differing opinions, the final recommendations offer flexible approaches to meet different levels of preparedness before the meeting. The recommendations are ready to be further tested as collaboration meetings are implemented more widely.

**Supplementary Information:**

The online version contains supplementary material available at 10.1186/s12913-026-14345-0.

## Background

Chronic pain significantly impacts the quality of life of those affected, influencing their participation in work, family life and leisure activities [1]. For chronic pain, a multidisciplinary approach, involving various services and professions, is often necessary [2]. However, this can lead to a high treatment burden [3], including challenges like receiving contradictory advice from different services, difficulties in finding available and affordable treatment options and interventions that are perceived to be accessible only to those who have the skills and energy available to fight for them [2, 4]. This highlights the necessity for interdisciplinary collaboration among healthcare providers to facilitate the establishment and management of treatment and care plans for individuals experiencing chronic pain.

Typically, most people with chronic pain are managed in primary care, whereas some may need specialised pain treatment at the level of secondary or tertiary care [5, 6]. The quality of the collaboration between primary and specialist care is thus important for these patients’ continuity of care, particularly because the general practitioner (GP) is the one who cares for the patient before and after specialist care. Such collaborations involve exchanging information, sharing insights and having a common understanding in order to work towards a shared goal based on what matters to the patient. However, poor communication between general practitioners and hospital specialists can challenge this and lead to consequences such as poorer quality, continuity and coordination of care [7]. Moreover, coordination is also challenging because of the different professional and financial frames in the various services [8]. For instance, barriers like lack of trust, communication and mutual understanding between professionals as well as incompatible organisational structures with differing aims can hamper collaboration and the coordination of care [9].

To overcome these challenges, collaboration through face-to-face meetings has been suggested due to their suitability for direct and synchronous information sharing, providing opportunities for trust building, and contributing to efficient decision-making [10]. However, it can be difficult to organize physical face-to-face meetings that include persons across care levels and from different sites [11]. For this, digital meetings in terms of videoconferences have been suggested. Previous studies on the use of videoconferences have reported benefits in patient care such as reduced travelling time and costs and convenience in consulting with clinicians [12]. However, there have also been described disadvantages of videoconferences regarding loss of richness of information due to a lack of nonverbal cues and that this can result in participants unconsciously using a more assertive communication style which again can have negative effects on collaborative discussions [11, 13].

Hence, collaboration between services is essential for the quality and continuity of care for persons with chronic pain but it also comes with some challenges. Collaboration meetings using digital solutions can be useful in overcoming these challenges. However, no studies on conducting digital collaboration meetings across healthcare levels and services for patients with chronic pain were found. Therefore, the aim of the study was to develop recommendations for how to plan and conduct digital collaboration meetings across care levels and services for patients with chronic pain.

## Methods

A qualitative study collecting data from a development process for digital collaboration meeting recommendations was conducted between October 2022 and June 2023.

### Setting

The setting of the current study is a tertiary pain centre at a university hospital in Central Norway which employs medical specialists, psychologists, physiotherapists, nurses and administration staff. In 2023, 673 unique adult patients (i.e., 18 years and above) with an average age of 42 years, were treated at the centre [14].

The Norwegian pain centres offer outpatient services on a tertiary or secondary service level in hospitals to people suffering from pain, regardless of diagnosis. The hospitals in Norway are managed by the regional health authorities. In Norway, potential patients are referred to the pain centres by their GPs or medical specialists. There are national guidelines for the pain centres given by the government, including criteria for whom to grant treatment [15]. The pain centres are multidisciplinary and employ staff representing various health professions with special interest and expertise in working with long-term pain, exemplified in the guide for the Norwegian pain centres with doctors from various specialities, psychologists/ psychiatric specialists, physiotherapists, and nurses [16].

Whereas the Norwegian hospitals are owned by the government and managed by the regional health authorities, the primary care services such as GPs, home care services and community physiotherapists are the responsibility of local authorities [17]. Moreover, all citizens in Norway are entitled to have a GP who is responsible for providing general health care, including follow-up after hospital admissions [17, 18].

The study is part of a larger innovation project on the implementation of a standardised care pathway extending into primary care for persons with chronic pain who have been given the right to access a tertiary pain centre. The background for the larger study was work on a national care pathway for persons with long-lasting and complex pain conditions led by the Norwegian Directorate of Health. A draft version of the pathway was sent out for public inquiry on the 10th of June 2022. The novelty of the draft was the provision of digital collaboration meetings in the pathway where the patient, the primary care professionals including the GP, and the pain centre professionals participate. According to the draft, the purpose of the digital collaboration meetings was to achieve a common understanding of a plan for follow-up and treatment between the patient, the pain centre, the regular GP of the patient and other relevant actors which aligns with what is important to the patient. According to the draft from the Directorate of Health, the collaboration meetings should be arranged for digital participation via video solutions to ensure efficient time use and reduce the treatment burden for the patient.

### Development process

In the process of developing recommendations for the digital collaboration meetings, the main activities were two pre-planned one-day workshops held in October 2022 and May 2023 with representatives of all relevant stakeholders for chronic pain care across services. The first workshop focused on what the digital collaboration meetings should be and how they should be organised. Based on contributions in the workshop, the first version of recommendations was made in January 2023. Data on the experiences with the preliminary recommendations were collected through meetings with pain centre staff who tried out digital collaboration meetings. Based on this, a second version of the recommendations was made. This second version was presented and discussed at a second workshop in May 2023 with the same stakeholders represented as in workshop one. The input from this workshop was used to make the final recommendations in June 2023, with the aim to be further tested as the digital collaboration meetings are implemented more widely.

The main activities in the final version of the recommendations are presented in Table 1 and in detail in Additional File 1.

**Table 1:** The main activities in the final recommendations on how to plan and conduct digital collaboration meetings

Phase/ activities	Recommendations
Professional preparations for the digital collaboration meeting:	The pain centre should conduct an interdisciplinary assessment, discuss possible interventions with the patient, create a comprehensive proposal for an action plan including follow-up and treatment, and clarify the patient’s preferences for the digital collaboration meeting.
Conducting the digital collaboration meeting:	The meeting should be led by pain centre staff unless otherwise agreed, and the patient’s wishes should guide who to invite to the meeting. The meeting consists of four parts: an introduction, a summary of the assessments, a review of interventions, making of an action plan, and a conclusion. If it is not possible to finalise the action plan, further follow-up should be scheduled to finalise the plan.
If staff from the Norwegian Labour and Welfare Administration (NAV) participate in the digital collaboration meeting:	Clarify with those involved how to handle sensitive topics, the duration of NAV’s participation in the meeting, and which NAV-related matters and interventions to discuss.
Template for an action plan:	The action plan should include what is important for the patient, current status, ongoing interventions, and future actions, specifying the area, description, rationale, responsibility, and timeframe.

### Participants

To develop the recommendations, the aim was to include three groups of participants: stakeholders, including patient representatives, involved in the care of patients with chronic pain, staff at the pain centre where the study originated, and researchers involved in the larger innovation project.

The stakeholders included healthcare professionals from various professions and different healthcare services, managers, administrative staff, patient organisations, health and social care authorities, and researchers. They were recruited by invitations to participate in the workshops that were broadly distributed by the project management to the relevant actors. The invitations included a description of the aim of the work, the draft of the standardised care pathway, information about the study and a consent form for data collection. The stakeholder groups in the two workshops are presented in Table 2.

**Table 2:** Participants in the workshops

Number/ Activity	Workshop 1	Workshop 2
Healthcare professionals, secondary and tertiary care	7	4
General practitioners	2	5
Other health professionals from primary care	5	4
Patient organisation representatives	3	2
Representatives from the Norwegian Labour and Welfare Administration	9	7
Representatives from the Norwegian authorities: the Directorate of Health and the Directorate of Labour and Social Inclusion	2	2
Researchers*	16	14
Other personnel (administrative support)	1	1

*Six of the researchers have combined positions as clinicians

The participants representing staff at the pain centre consisted of those who participated in the pain centre’s regular meetings where suggestions for recommendations were discussed. The pain centre staff were from various professions such as psychologists, physiotherapists, nurses, medical specialists and administration.

The researchers involved in the larger innovation project included researchers from the university and the hospital with expertise in various health sciences and health services research. All members of the author group were part of the research team. In addition, all members of the author group participated in the workshops as well as in several of the pain centre staff meetings.

### Data collection

Data were collected from workshops, the pain centre staff meetings and the meetings in the research group (Table 3). In brief, the workshops included short lectures, group work, and activities such as poster-making and polls. Examples of questions raised in the workshops were: “What, from the already existing collaboration between the primary care and specialist services, would you suggest should be used to improve the collaboration and/ or what should be preserved?” and “Which of the principles- being patient-centred, considering quality of life and coping, working in an interdisciplinary team and engaging in shared decision-making- do you think are most important for the collaboration meetings?”. From pain centre staff meetings and the meetings of the researchers, the data collected included observations, reports and summaries from the discussions on the suggested content in the recommendations and their experiences of trying out digital collaboration meetings. These activities were carried out before, between and after the workshops.

**Table 3:** Overview of activities, participants and collected data

Activity	Aim of activity	Participants and data	Settings represented
Workshops	Exploring stakeholders’ needs and views related to content in the recommendations	45 participants in workshop 1 and 39 participants in workshop 2. In total, 48 pages of written responses and 6 posters from group work and discussions.	Primary care Secondary and tertiary care National government Patient representatives Norwegian Labour and Welfare Administration (NAV) Research
Pain centre staff meetings	Feedback on suggested content and shortcomings in the recommendations	Physicians, nurses, physiotherapists, psychologists, and administrative staff, on average 12 participated in 6 meetings. In total, 6 pages of written material from observations and summaries.	Tertiary care Research
Research team meetings	Develop and adjust the recommendations	Researchers, healthcare professionals, NAV representatives, and pain centre management, on average 13 participated in 30 meetings. In total, 30 pages of written reports.	Research Tertiary care NAV

### Data analysis

The analysis was an ongoing iterative process conducted during the process of developing the recommendations, with a last round of analysis done after the final recommendations were made. During the development of the recommendations, the focus of the analysis was mainly on giving input to the recommendations, while in the final round, which is presented as the results, the focus was on the topics that generated the most discussions during the development of the recommendations.

The data were analysed using a practical thematic analysis approach [19], with three steps that were done iteratively. The first step concerned ‘Reading’. All authors immersed themselves in the collected data to become familiarised with the full dataset and get initial ideas. The data material was then divided between the authors to write individual summaries and memos that presented initial ideas to be discussed in the author group.

The second step was ‘Coding’. All authors coded separate parts of the data material in pairs. The codes were compared and discussed with the others in the author group. Examples of codes were the aim of the digital collaboration meeting, technical versus relational issues and trust.

The third step concerned ‘Theming’. In the final round of analysis, two analysis sessions were held with all authors. In these sessions, perspectives were shared and discussed among the authors. Themes relevant to inform the development of the recommendations were discussed, and a set of final themes was developed. Based on this, the first author drafted the final report of the results which were repeatedly discussed in the author group until agreement was reached.

## Results

The data were categorised into three themes that highlight the most prominent discussions during the development of the recommendations: “Patient-centredness in the digital collaboration meeting”, “A common action plan” and “Good meeting climate”.

### Patient-centredness in the digital collaboration meeting

Both in the workshops and other discussions, there was an agreement that the leading principle for the digital collaboration meetings should be “*the patient at the centre*”. Nevertheless, several of the discussions were about how to understand what “patient-centred” means in practice and what this entailed for the digital collaboration meetings. A common saying among the professionals, was that being patient-centred was about safeguarding the patient-professional relationship, establishing trust, and being aware of the balance of power in meetings between patients and professionals. Among some of the researchers, it was talked about patient-centredness as ensuring that the wishes of the patient are taken into account and that the patient is involved enough to get an understanding of the situation and is given the possibility to be involved according to their preferences.

In the first version of the recommendations, a patient-centred orientation was made visible by writing that for all topics addressed in the digital collaboration meeting, the patient should speak first and always be given the opportunity to comment on what was presented by the clinicians in the meeting. One challenge raised in discussions following the first version of recommendations was that not all patients were necessarily comfortable with speaking in meetings involving many participants. Another issue brought forward by patient representatives was that it could be demanding for the patient to take in the results of the pain centre assessments while at the same time participating in discussions on possible interventions and actions. Based on the discussions, from the second version of the recommendations, it was specified that the patient should be allowed to speak first, but that there should be no requirement for the patient to say anything.

Another topic raised several times related to patient-centeredness, was whether healthcare professionals from the pain centre and the GP should discuss assessments and possible interventions before the digital collaboration meeting without the patient being present, or whether being patient-centred meant that the patient should be part of all discussions concerning their situation. Arguments for the separate meetings without the patient being present were that it was important that the professionals should resolve disagreements and should present a united front when presenting results to the patient. This would help in reassuring the professionals when they knew what the other professionals thought, and it reassured the patient that the professionals agreed.

All health professionals involved must be well informed, work together and speak the same language” (Written response in Workshop 1).

Arguments for including the patients in such discussions were that leaving them out meant the professionals were more prepared than the patients for what was to be discussed in the meeting. In a pain centre staff meeting, this was summarised as being about balancing the power in the collaboration meeting by giving the patient an active role versus the need for the professionals to be able to work interdisciplinary in an efficient way. Due to the differences in opinions, the issue was omitted from the latest version of the recommendations. Thus, the recommendations do not provide guidance either for or against including the patient in all discussions concerning their care and treatment.

### A common action plan

As it was stated in the draft from the Directorate of Health on the standardised pathway, it was clear from the onset that a common action plan should be a result of the digital collaboration meeting. This was also agreed on in general by the participants and it was commented to be common clinical practice to make plans for what to do next. However, the process of developing the recommendations showed that there were both professional opposition to the suggested solution for making a common plan and that there were practical issues that were not easily solved.

The usual procedure at the pain centre was that the patient had consultations with different clinicians individually before the clinicians discussed the patient and informed the patient about their suggestions in a joint meeting. To maintain this workflow while simultaneously facilitating the intention of the digital collaboration meeting of including GPs and others, the first version of the recommendations for the digital collaboration meetings said: “*Create a comprehensive proposal for a plan for follow-up and treatment and distribute it before the digital collaboration meeting*”. The argument for this was that everyone participating in the meeting should have the opportunity to know which actions would be discussed. It was also intended that the professionals together with the patient should make suggestions for actions to be included in the plan in the individual consultations and that one clinician at the pain centre should summarise these into a final suggestion before the digital collaboration meeting. A template for the suggested action plan was made to ensure that everyone would have access to the same minimum amount of information needed to understand the suggestions. The suggested template was kept unchanged in the three versions of the recommendations.

However, a practical issue that quickly arose concerning distributing an action plan before the digital collaboration meeting, was that there was no system in place for sharing suggestions with others, neither was there any allocated time for this, and doing so would mean less time for other clinical work at the pain centre. Furthermore, some clinicians argued that such a focus on actions would not fit with a frequently occurring situation they had at the pain centre where the main thing to do for a patient would be to remove some of the ongoing activities and calm down the situation. However, the main professional argument against the pre-meeting distribution of an action plan was that many clinicians said they needed to talk to the other clinicians before they made suggestions. It was also said that many clinicians were not used to discussing possible actions directly with the patient before they talked to their colleagues.

The reports from those trying out the digital collaboration meeting were that pre-meeting distribution of a suggestion for a plan was difficult, and in practice not done. It was especially challenging to find enough time before the collaboration meeting to talk to the other clinicians about further actions. Consequently, the digital collaboration meetings were organised with the first 15 min being allocated for the clinicians from the pain centre to talk to each other, and if found appropriate, the GP. After they finished, the patient was invited to join the meeting, and 30 min were allocated for this. If issues related to work and/or social security benefits were present, the case handler from the Norwegian Labour and Welfare Administration (NAV) participated during the last 15 min. To accommodate this practice, while still highlighting the original intention of distributing a suggested plan before the digital collaboration meeting, two options on how to conduct the meeting were described. Both were based on the second version of the recommendations. One alternative was to be used if a suggested plan had been made and ideally distributed beforehand and the other option was to be used if not. For the latter case, the recommendation was to “*Achieve common understanding about further work with a plan for follow-up and treatment that supports what is important to the patient*”. Hence, the initial proposal of making a suggested plan as part of the preparation for the digital collaboration meetings was kept in the final version of the recommendations because it was seen as an important activity to fulfil the intention of the digital collaboration meeting being a meeting where the plans for further actions were agreed upon.

Based on input in the workshops, it was written in the recommendations that the action plan should be based on which goals were important to the patient. An argument put forward for this was that it is the patient who must implement the action plan, and it is therefore important that the actions are aligned with what the patient considers important outcomes. One concern raised was that it could take time for the patient to become confident enough to formulate expectations and wishes and to become aware of what opportunities existed. However, it was also discussed among the healthcare professionals that challenges could arise when the patient’s wishes were something other than what the professionals considered realistic or even desirable. One example mentioned was whether it could create unrealistic expectations if it was perceived by the patient as completely open what the outcome of the collaboration meeting could be.

*What happens when professional knowledge and evidence do not match what the patient wants? Which tools/principles should then be used*? (Written response in Workshop 2).

### Good meeting climate

To make the digital collaboration meeting a good experience for those involved, two issues were specifically discussed: meeting management and the role as chair of the meeting.

Good meeting management was consistently seen as important for succeeding with the digital collaboration meeting. Suggestions for how to ensure a good meeting included having an agenda so that everyone knew what was to be talked about, that necessary clarifications were made at the start of the meeting, having a summary of conclusions from the meeting and ending up with a plan for the time ahead. Moreover, it was emphasised as essential that everyone had the opportunity to say what they wanted to say in the meeting.

There was no opposition to arguments put forward that a good meeting depended on having a chair, but there were discussions on who should be the chair leading the meeting. It was suggested in the first version of the recommendations that *“The patient is asked who s/he thinks should lead the meeting*”. The rationale for this was that there could be situations where the patient had a strained relationship with some of the participants in the meeting and thus should have the opportunity to influence who led the meeting. Both in the workshops and discussions at the pain centre, some statements showed that this recommendation was perceived as the patient deciding who should lead the meeting. Moreover, some spoke about problems arising if someone else than a representative of the pain centre led the meeting, as they would lack the full overview. In the latest version of the recommendations, it was therefore formulated as “*Someone from the pain centre leads the meeting unless otherwise agreed*”.

Finally, there were discussions regarding where the patient should be during the digital collaboration meeting. It was argued that it had to be ensured that the patient was taken care of and had support during the meeting. Some argued that the patient should be with their GP because the GPs were the ones to follow up on the action plan together with the patient. Others argued that because the GPs often did not have equipment that allowed both the patient and the GP to be visible on the screen, it was better that the patient was at the pain centre. Others argued that for some patients, the best thing would be to participate in the meeting from home with their next of kin. Based on the different views on the matter at the same time as emphasizing a patient-centred approach, the final version of the recommendations stated that the patient should decide for themselves where to attend from but always be offered to be with healthcare professionals.

## Discussion

A set of recommendations for how to plan and conduct digital collaboration meetings across care levels and services for persons with chronic pain was developed and agreed upon. However, the final version does not include advice on whether to involve the patient in all discussions and has two options for the process of making an action plan. The reason for this were disagreement and practical challenges regarding what it meant to place the patient at the centre of the activities, whether the patient should be present when professionals discussed the interprofessional assessments, whether clinical notes and suggestions for action should be distributed before the meeting, and how to ensure well-led meetings with clear agendas.

The most frequently discussed topic during the development of the recommendations was the implications of being patient-centred in digital collaboration meetings. Being patient-centred entails placing the patient’s beliefs and values at the core of decision-making and treating the patient as individuals and equal partners [20, 21]. This principle was in the workshops unsurprisingly considered the most important one for planning and conducting digital collaboration meetings. However, several discussions arose when developing recommendations that aimed to put this principle into practice.

A Swedish model for person-centred care for individuals with chronic pain summarizes patient-centredness in three steps: (1) Initiating personal narratives to get to know each patient as a person and to identify their previous experiences, present situation, needs, abilities, and resources, (2) Co-creating a personal plan in line with identified resources and barriers combined with medical, health, and social research evidence, and (3) Documenting and monitoring the plan and adapting it to changes in the goals and/ or other circumstances [22]. The developed recommendations correspond to these steps and hence, align with the evidence in the field of patient-centred pain care. While few studies address patient-centredness in collaboration meetings for individuals with chronic pain involving healthcare professionals across services, a recent systematic review on oncological multidisciplinary team meetings highlights that, despite the benefits, patient-centredness often receives insufficient attention [23]. Our results support this, and discussions during the development of the recommendations indicate that, although the principle of being patient-centred is widely acknowledged, discussing its practical application among those involved in the care is valuable and often necessary. Notably, whether digital meetings are better for the patient in contrast to physical meetings, other than from a cost-time perspective, remains to be explored.

One key discussion was on whether the patient should participate in all discussions concerning their care or whether the GP and pain centre staff should have a pre-meeting without the patient present. The principle of “Nothing about me without me” has often been linked to patient empowerment and patient-centred care in many countries, including Norway [24]. If followed to the letter, the principle would imply that the patient should participate in every discussion that concerns their situation and care. In the current study, some of the opposition to this was linked to that the professionals needed to clarify their assessments and suggestions for further action before entering the collaboration meeting to avoid disagreements in the meeting and causing anxiety in the patient. The raised concerns are in line with previous studies on the implementation of patient-centred care [25] and from having the patient present at oncological multidisciplinary team meetings [23].

Another key discussion on the practical implications of being patient-centred was related to who should lead the digital collaboration meeting. Following the patient-centred principle, it seems reasonable that the patient should have a say in who leads the digital collaboration meeting. However, professionals opposed this, fearing the patient might choose someone outside the pain centre, which could cause practical challenges. At the same time, it was emphasised that the meeting should be led in a way that ensured a good climate. One could argue that, in order to achieve this, it is important that the patient has trust in the person leading the meeting. The professionals’ opposition was related to ensuring that the chair of the meeting was able to lead the meeting in a good way. Hence, it seems that the opposition to the patient choosing whom to lead the meeting was more connected to uncertainty on how this would play out than to restrict the patients’ involvement. However, as pointed out by others [26], more evidence is needed to understand the experiences of engaging patients in such processes and whether this translates into enhanced care processes.

Regarding previously described barriers to continuity of care like lack of communication and mutual understanding between professionals [9], it can seem reasonable that healthcare professionals across the involved services coordinate their suggestions for future care before meeting the patient. Also, patients have reported that harsh communication between professionals in meetings induces daunting feelings [27]. This indicates that, if there are disagreements, it might be better to address them without the patient being present. However, promoting an active patient role requires that the patient has access to information and is involved in decisions. Moreover, ensuring that the plan for follow-up and treatment aligns with what is important to the patient requires patient participation. Hence, there are several considerations related to including the patient in all discussions and due to the differences in opinions among the stakeholders in the study, the issue was omitted from the latest version of the recommendations, and it is up to the individual healthcare professional to decide whether to arrange pre-meetings before the digital collaboration meetings.

Although not explicitly talked about, the financial and resource constraints that the digital collaboration meetings are to be conducted in, require efficient use of resources. The choice of using video conference solutions for the collaboration meetings was not questioned, nor were challenges with poorer communication talked about. However, it was mentioned that the necessary equipment for conducting digital collaboration meetings was not always available, especially in the GP offices. In addition, the professionals said they lacked solutions for easy sharing of suggestions for an action plan. Although the use of technology in healthcare has rapidly increased in recent years, our study is in line with others [11, 13] in that available equipment and solutions of high quality must be available to be able to achieve the goals of interventions such as collaboration meetings across healthcare levels for patients with chronic pain. Moreover, future studies should investigate whether using digital solutions for collaboration meetings is appropriate for efficient and qualitative care.

Despite the challenges discussed here, for the most part, there was agreement on the recommendations. One reason is likely that involving all stakeholders relevant to the digital collaboration meetings enabled a common process where all voices were heard. Previously described barriers to the organization of care for chronically ill persons such as lack of trust and lack of communication between professionals [9], were not evident in the results. Nevertheless, the discussions on how to establish a good climate and balance the power in the digital collaboration meeting emphasise the importance of mutual trust, transparency in decisions, sharing of insights and having joint discussions on the best way forward, as described previously [7]. The engagement in the process and the fact that not all discussions were concluded can be viewed as an argument for having an ongoing process when changing practices and routines. Nevertheless, involving all stakeholders in a common process was fruitful and provided a good starting point for implementing and further developing the recommendations.

### Strengths and limitations

The main strength of the study was the broad involvement of stakeholders and the iterative process of developing the recommendations for conducting digital collaboration meetings. However, there are some noteworthy limitations. First of all, there was no direct observation of the digital collaboration meetings where the recommendations were tested, and information on this relied on the reports of the clinicians. Although both positive and negative things were said about the suggested recommendations, it might be that the clinicians adapted their stories to serve their viewpoints. No patient representatives were included as members of the research group. However, they participated in the workshops in which preliminary findings were presented and discussed. Consequently, patient representatives played an important role in shaping the recommendations. Nonetheless, it is possible that the final formulations might have differed had they also been part of the research team. Furthermore, despite the technical challenges of pre-meeting distribution of information, it was not developed recommendations for how to handle this. Moreover, the recommendations do not address technical challenges for videoconferencing, as these will depend on local conditions. Nevertheless, the technical part of the collaboration meetings is an important area and should be followed up with local recommendations. Finally, because the findings are based on a study in a Norwegian healthcare setting, the findings are mainly generalisable to other settings with similar organisations. Still, the findings will likely be valid for digital collaboration meetings in other types of settings, as they concern some common issues regarding continuity of care and patient-centredness.

## Conclusion

A set of recommendations for how to plan and conduct digital collaboration meetings across care levels and services for persons with chronic pain was developed. Due to practical issues and differing opinions, the final recommendations offer flexible approaches to meet different levels of preparedness before the meeting and are ready to be further tested as digital collaboration meetings are implemented more widely. Future research should investigate both the advantages and disadvantages of digital collaboration meetings, in addition to how digital collaboration meetings are experienced and if they provide the expected benefits.

## Supplementary Information

Below is the link to the electronic supplementary material.

Supplementary Material 1

## Data Availability

The datasets generated and analysed during the current study are not publicly available due to data regulations but are available from the corresponding author upon reasonable request.
