# Supplementary material for: Developing recommendations for digital collaboration meetings across healthcare levels and services for patients with chronic pain- a qualitative study
Source: BMC Health Serv Res. 2026 Mar 11;26:425. doi: 10.1186/s12913-026-14345-0 (PMC13032243; doi:10.1186/s12913-026-14345-0)
Supplement: Supplementary file 1 — Supplementary Material 1 [file 12913_2026_14345_MOESM1_ESM.pdf]

Recommendations on how to conduct digital collaboration meetings

Professional preparations at the pain centre before the digital collaboration meeting

1. Complete a multidisciplinary assessment and discuss possible interventions to be included in an action plan with the patient
    a. Inform the patient about the standardised care pathway and the collaborative meeting
    b. Clarify factors of importance for the patient in the short and long term
    c. Carry out the assessments and the documentation, including a summary that can be included in the first part of the action plan

2. Make a common proposal for an action plan and distribute it to all participants in the meeting
    a. One responsible person from the pain centre collects suggestions for all parts of the action plan and makes a first draft/proposal for a common plan
    b. Send the first proposal of the action plan to all participants of the collaborative meeting

3. Clarify the patient's preferences for the digital collaboration meeting regarding the following:
    a. Which persons does the patient want to include in the digital collaboration meeting. The clinicians may want to suggest some persons for the patient to bring.
    b. Where does the patient prefer to be located during the digital collaboration meeting (at home, at the pain clinic, at the general practitioner's office, or other locations)
    c. The patient preference of the person to be the leader of the meeting (the patient, the patient's coordinator, one of the pain clinic clinicians, the general practitioner, and others)

The carrying out of the digital collaboration meeting

1. Meeting leadership and introduction
    a. All the invited persons (including the patient and the persons the patient wishes to include) will participate throughout the meeting so that all participants are involved in all the discussions and decisions.
    b. If staff from the Norwegian Labour and Welfare Administration (NAV) participates in the digital collaboration meeting: Clarify with those involved how to handle sensitive topics, the duration of NAV's participation in the meeting, and which NAV-related matters and interventions to discuss.
    c. The digital collaboration meeting is led by pain clinic personnel if otherwise has not been agreed with the patient.
    d. The leader of the digital collaboration meeting must actively strive to create a safe atmosphere and make sure that all participants get to speak their minds.
    e. Start with a short round of introduction, the name and role of the participants

f. Give the patient an opportunity to convey what he/she wishes to get out of the digital collaboration meeting.
g. Clarify the purpose of the digital collaboration meeting: to gain a common understanding of a plan for further follow-up with interventions that support factors that are important to the patient in the short and long term.
h. Clarify who should be responsible for notifying of changes in the follow-up plan during the meeting.

2. Summarizing the multidisciplinary consultations
    a. Give a summary of findings from the clinical assessments at the pain clinic
    b. Questions and input to the summary of findings (the summary constitutes the initial part of the plan: What is important to the patient / Status / Ongoing interventions). Clarify uncertainties.

3. Work through interventions OR suggest interventions if the proposal for an action plan has not been completed
    a. Consider the proposed or suggested interventions consecutively:
        i. The pain clinic clinician presents the proposed intervention
        ii. The patient is given the opportunity to comment on the proposed intervention before the comments from the other participants
        iii. Discussions and clarifications of each proposed intervention
    b. Ask the patient if he/she would like to summarise his/her understanding of all the interventions

4. Ending the digital collaboration meeting
    a. Agreement on further action plan and evaluation, including agreement on time and date for a second collaboration meeting within 6 months.
    b. Evaluate the collaboration meeting. Ask the patient about his/ her experience of the meeting.
